# Microneedle patch delivery of influenza vaccine during pregnancy enhances maternal immune responses promoting survival and long-lasting passive immunity to offspring

**DOI:** 10.1038/s41598-017-05940-7

**Published:** 2017-07-18

**Authors:** E. Stein Esser, Joanna A. Pulit-Penaloza, Haripriya Kalluri, Devin McAllister, Elena V. Vassilieva, Elizabeth Q. Littauer, Nadia Lelutiu, Mark R. Prausnitz, Richard W. Compans, Ioanna Skountzou

**Affiliations:** 1 Department of Microbiology & Immunology and Emory Vaccine Center, Emory University School of Medicine, Atlanta, GA 30322 USA; 2 School of Chemical and Biomolecular Engineering, Georgia Institute of Technology, Atlanta, GA 30332 USA

## Abstract

Influenza virus causes life-threatening infections in pregnant women and their newborns. Immunization during pregnancy is the most effective means of preventing maternal and infant mortality/morbidity; however, influenza vaccination rates of pregnant women remain under 50%. Furthermore, the availability of vaccines in low-resource populations is limited. Skin immunization with microneedle patches (MN) is a novel and safe vaccination platform featuring thermostable vaccine formulations. Cold-chain independence and the potential for self-administration can expand influenza vaccination coverage in developing countries. In this study of pregnant BALB/c mice immunized with subunit H1N1 influenza vaccine, we demonstrate the advantage of skin vaccination over intramuscular delivery of a two-fold higher vaccine dose. MN vaccine induced superior humoral immune responses and conferred protective immunity against a lethal challenge dose of homologous influenza virus. Importantly, MN vaccination of mice at mid-gestation resulted in enhanced and long-lasting passive immunity of the offspring, measured by neutralizing antibody titers and survival rates after virus challenge. We conclude that skin vaccination using MN is a superior immunization approach with the potential to overcome immune tolerance observed in pregnancy, and lower vaccination costs through antigen dose-sparing, which is especially relevant in underserved countries.

## Introduction

For nearly a century, the immunotolerant status during pregnancy has been an acknowledged risk factor for severe complications from various infectious agents due to reduced immune responses to antigens. Influenza infections during the second and third trimester of pregnancy cause up to fivefold increases in cardiopulmonary complications compared to a non-pregnant population^1^. The risk of influenza-associated hospitalization and mortality increases as the pregnancy progresses^2^. Mortality rates were highest among pregnant women (as high as 45%) during major influenza pandemics (1918, 1957, 1968 and 2009)^3^. Infection-related complications extend to the fetuses and neonates, with well-known increased risk of miscarriage, stillbirth, neonatal death, preterm birth, and low birth weight neonates^1, 3–8^.

For safety reasons, influenza vaccines are not licensed for use in infants less than 6 months old. Therefore, the most effective way to protect embryos *in utero* or newborns postnatally from adverse effects of influenza infection is through vaccination of pregnant women. Efficacy of influenza vaccines in mothers and infants has been evaluated in several human studies with varying outcomes (reviewed in ref. 9). Those studies which are based on laboratory-confirmed cases or clinically diagnosed influenza infection have reported that vaccination during pregnancy reduced the risk of influenza infection by approximately 70% and the risk of preterm birth by 37% compared to non-vaccinated pregnant women^10, 11^.

Flu vaccine is recommended for administration to unvaccinated pregnant women in the late second or third trimester (after 20 weeks gestation) for two reasons: a) the current subunit or split influenza vaccines induce a fairly short-lived immunity, with antibody titers waning after 6–7 months post-vaccination, so that a late vaccination would successfully protect the mother until labor, and b) since infants are not vaccinated against influenza before 6 months of age, it is desirable to confer a robust passive immunity to them by transplacental transfer of maternal antibodies while *in utero* or by breast milk during the nursing period of the infant. Influenza vaccines given to pregnant women can be up to 91.5% effective in preventing influenza-related hospitalization of their infant children at 6 months or younger^12^.

In recent years, the World Health Organization’s (WHO) Expanded Program on Immunization Practices recommended influenza vaccination for all pregnant women regardless of pregnancy trimester, as well as for women of childbearing age^13, 14^. Despite the more relaxed immunization timelines, only 50% of women in the U.S. were vaccinated either before (15.3%) or during pregnancy (35.0%) in 2015^15^. Major bottlenecks for the implementation of influenza vaccination programs in developing countries include the lack of access to health care services, as well as shortages in trained health care personnel. Other logistical and economic obstacles are vaccine cold chain requirements with increased costs, ineffective immunization campaigns due to lack of information or socioeconomic factors, and needle-phobia^16^.

We have previously demonstrated in animal models that skin immunization with influenza vaccine using microneedle patches (MN) induces potent and longer-lasting immune responses as compared to conventional vaccination with needle and syringe^17, 18^. In addition other investigators have reported that IM or intraperitoneal (IP) influenza vaccination of pregnant mice protects them and their fetuses from influenza infection^19, 20^.

MN are patches containing micron-scale, solid needles made of biocompatible, water-soluble materials encapsulating vaccines, drugs or other compounds of interest^21–24^. MN can be painlessly applied to the skin by minimally trained personnel or patients themselves^25–27^. The microneedles dissolve in the skin, leaving no biohazardous sharps waste^16^. Thermostability of MN vaccines has been shown, including stability of influenza vaccine for at least two years at 25 °C^28^. By targeting Langerhans cells, dermal dendritic cells and other antigen-presenting cells in the skin, immunological advantages have been shown for skin vaccination using MN with a number of different vaccines^21–24^, including influenza^29–33^. Excellent safety, immunogenicity and acceptability of MN for influenza vaccination in a phase I clinical trial was recently reported^34^.

Based on these findings, we fabricated MN to deliver subunit seasonal influenza vaccine in the skin of pregnant BALB/c mice at mid-gestation. The goal of this study was to determine for the first time whether influenza vaccination with MN could overcome the immune tolerance during pregnancy by harnessing the innate immune cell machinery of the skin and confer robust immunity to mothers and their offspring. We also compared the MN-induced immune responses to those elicited by the conventionally used vaccine delivery method, intramuscular (IM) vaccination or another cutaneous approach; intradermal injection (ID).

## Results

### MN patches showed successful skin penetration and antigen release

**Figure 1:**
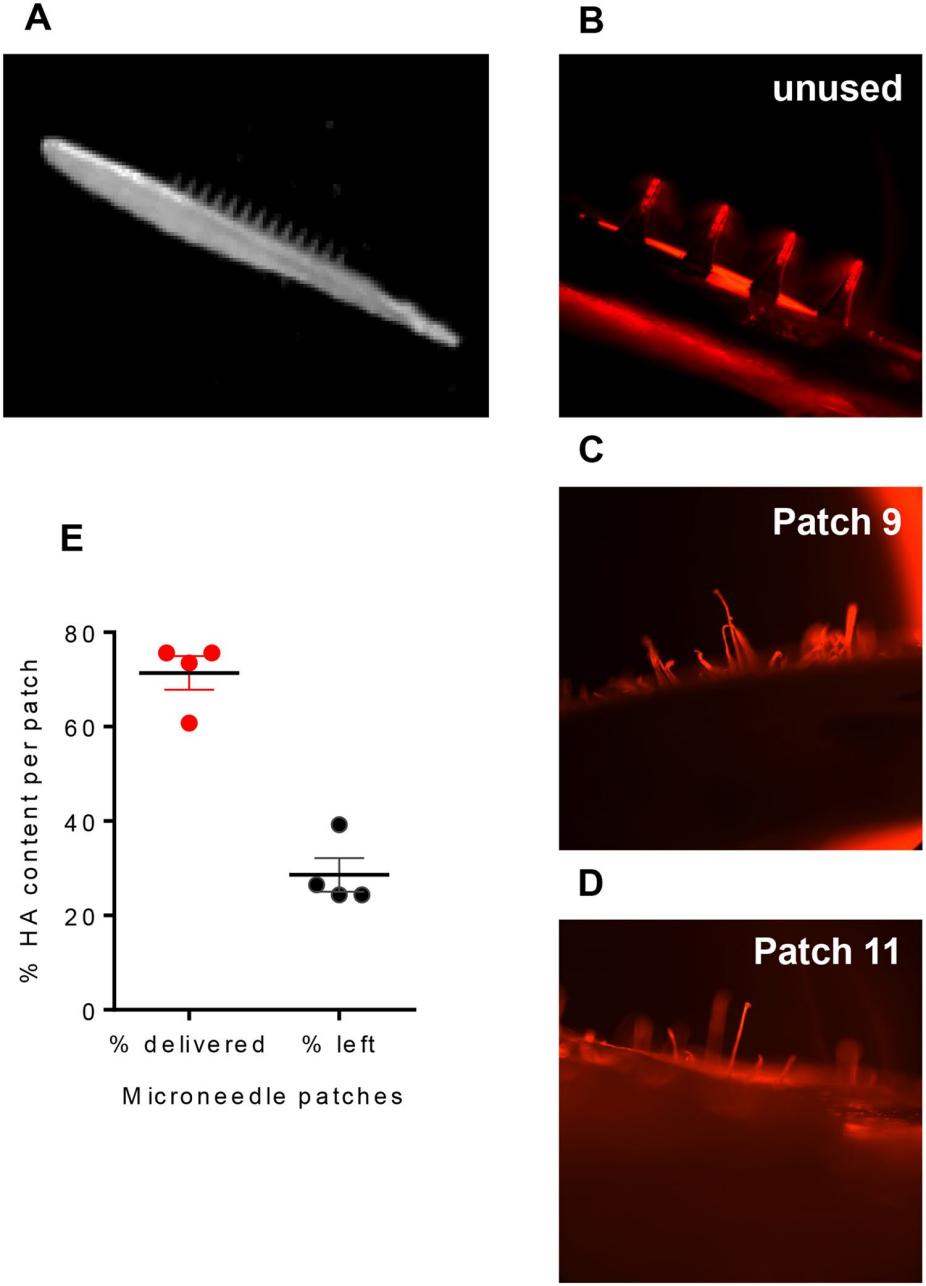
Encapsulation and Delivery of influenza vaccine using dissolving MN patches. (**A**) Microneedles were loaded with high dose of A/Brisbane/59/07 subunit vaccine (15 μg). The patches were sealed with desiccant and kept @ +4 °C. After being brought to the room temperature and unsealed, MN retained their structure and rigidity. (**B**) Unused MN patches were imaged with inverted microscopy (20x magnification). (**C,D**) Used MN patches 10 minutes after insertion into murine skin (20x magnification, inverted microscopy). Some stretched out protrusions can be seen in the used patches possibly containing undelivered vaccine. (**E**) Percent residual A/Brisbane/59/07 vaccine per patch after insertion into murine skin. The vaccine content after loading and after insertion was determined by SRID. The vaccine was extracted from the patches in the same volume of PBS and run in triplicates. SRID standards ranged from 10 to 70 μg/ml HA. The values were expressed as mean ± SD.

MNs containing influenza vaccine (Fig. 1) were prepared with sulforhodamine in order to visualize the microneedles. The microneedles showed successful penetration and dissolution in mouse skin within 10 min (Fig. 1C and D). On average we observed approximately 10% losses in antigen loading per patch; although we targeted to encapsulate 15 μg of HA, SRID data showed an average of 13.49 ± 1.32 μg HA (average ± standard deviation, n = 4) loading (Table 1). After insertion in the skin, the residual material was 3.86 ± 1.03 μg, indicating a delivery efficiency of 71% (Fig. 1E).

**Table 1:** Delivery efficiency of FG patches.

Patch #	HA in fresh patch, μg	HA in used patch, μg	HA, % remaining
9	15.30	3.73	24.38
10	13.64	5.35	39.22
11	12.78	3.39*	27.52
12	12.27	2.99*	24.37
Av ± SD	13.49 ± 1.32	3.86 ± 1.03	28.62

*Slightly below lowest STDEV.

### Vaccination of animals at mid-gestation

Following immunization of pregnant mice, body weights were recorded daily until delivery. Similar to our previous observations made with tetanus vaccine in pregnant mice^35^, no adverse effects of IM, ID or MN vaccination on the outcome of pregnancy were observed (i.e. body weight fluctuations or preterm labor).

### Vaccination of pregnant mice induces lower humoral immune responses than those in non-pregnant mice

**Figure 2:**
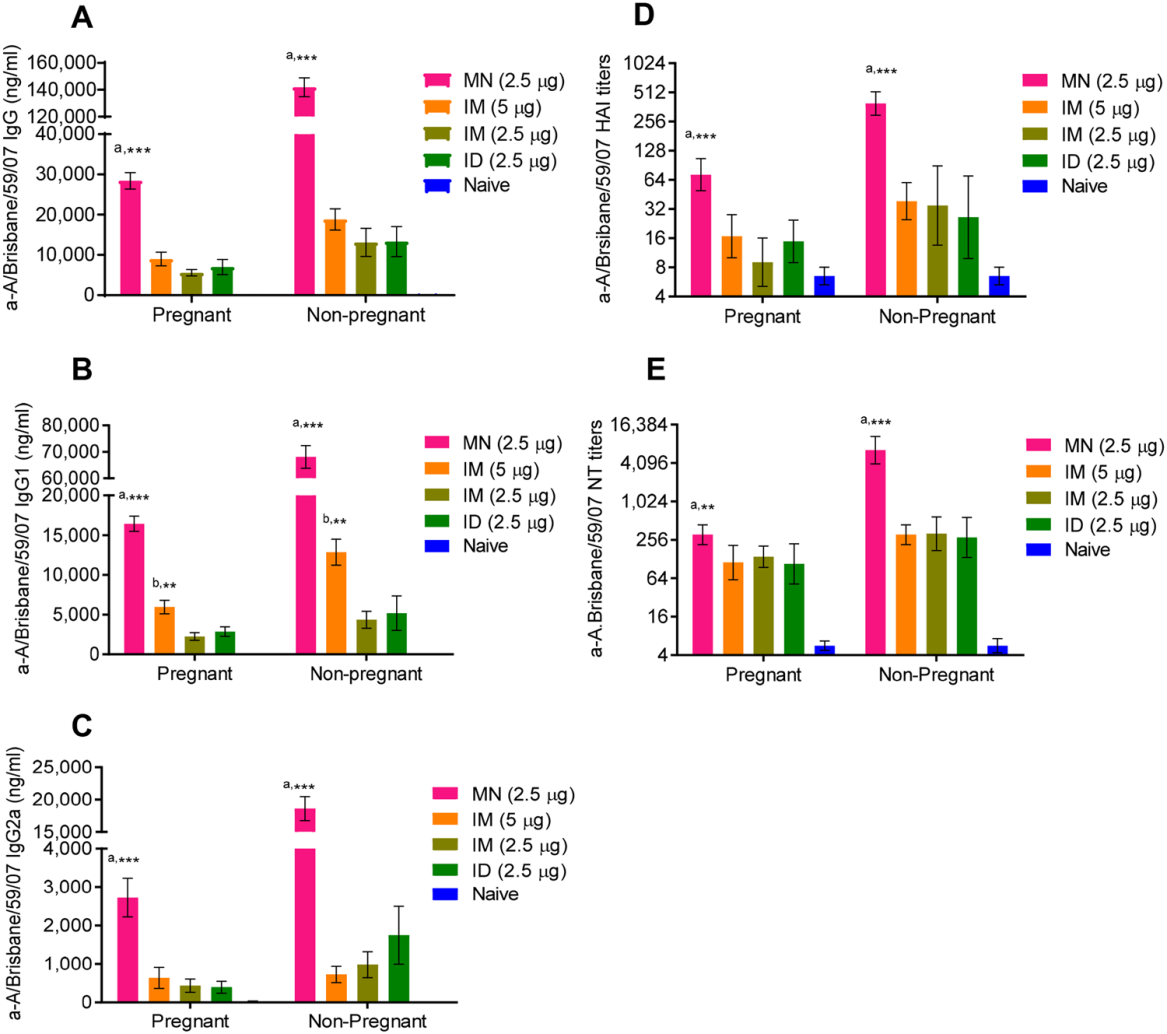
Humoral immune responses in pregnant mice immunized with A/Brisbane/59/07 H1N1 subunit vaccine via intramuscular or transcutaneous routes. Anti-influenza binding antibodies were determined by ELISA in sera collected from mice 28 days after immunization. (**A**) IgG (**B**) IgG1 and (**C**) IgG2a antibody titers. Values are expressed as mean ± SEM, (n = 5–20). (**D**) ^†^Hemagglutination inhibition (HAI) and (**E**) neutralizing antibody (NT) titers in sera collected 28 days after immunization. Values are expressed as geometric mean with a ±95% confidence interval (n = 5–20). ^†^Naive: unimmunized mice; IM: vaccine administered intramuscularly; ID: vaccine administered intradermally with syringe and needle; MN: microneedle patches encapsulating the vaccine. ^†^[Pregnant mice: MN (2.5 µg), n = 14; IM (5 μg), n = 8; IM (2.5 μg), n = 5; ID (2.5 μg), n = 5; naïve, n = 5. Non-pregnant mice: MN (2.5 µg), n = 20; IM (5 µg), n = 20; IM (2.5 µg), n = 5; ID (2.5 µg), n = 5; naïve, n = 5]. ^a^[MN *vs* IM (5 μg), IM (2.5 μg), and ID (2.5 μg)]. ^b^[IM (5 μg) *vs* IM (2.5 μg), and ID (2.5 μg)]. *p < 0.05; **p < 0.01; ***p < 0.001; ****p < 0.0001. Data were analyzed with Mann-Whitney unpaired non-parametric t-test.

We observed that a single dose of influenza subunit vaccine induced 2–5 fold lower serum IgG, IgG1 or IgG2a titers in IM, ID or MN immunized pregnant mice when compared to non-pregnant mice (Fig. 2A–C). The reproducibility of these observations corroborates with reports by other investigators on the role of pregnancy hormones in suppression of the immune response^36–38^.

### MN immunization conferred superior humoral responses compared to ID or IM immunizations in both pregnant and non-pregnant mice

Non-pregnant mice immunized with MN significantly outperformed all other groups in the extent of their immune responses as early as one month post-vaccination (Supplementary Table 1), showing 10-fold higher vaccine-specific IgG serum antibody titers than non-pregnant mice vaccinated IM with the same vaccine dose (p < 0.0001) and 7.5-fold higher vaccine-specific IgG antibody titers than mice immunized IM with a two-fold higher dose of the vaccine (p < 0.0001) (Fig. 2A). The difference between MN and IM vaccinated groups was even more pronounced in IgG2a titers (25-fold higher), and it was 10-fold higher when comparing MN and ID groups (p < 0.001), showing that skin vaccination with either microneedles or intradermal needle improves the Th1 responses (Fig. 2C).

Microneedle immunization was also found to confer superior immune responses in pregnant mice compared to IM immunization. Mice immunized with low dose of subunit vaccine (2.5 µg HA) via MN, had IgG, IgG1, and IgG2a antibody titers about 3–4 fold higher than pregnant mice immunized with 5 µg of HA via the standard IM route (p < 0.001) and 4–7 fold higher than pregnant mice immunized with 2.5 µg of HA via the IM or ID routes (p < 0.001) (Fig. 2A–C) (Supplementary Table 1). Overall, these data provided further evidence on the role of pregnancy in shaping the magnitude and quality of humoral immune responses, although both pregnant and non-pregnant animals showed dose-dependent antibody responses. Most importantly, MN delivery enhanced these responses and showed significant dose-sparing as this route outperformed the standard IM approach using twice-higher vaccine dose.

### MN patches induced superior influenza-specific functional antibody titers

**Figure 3:**
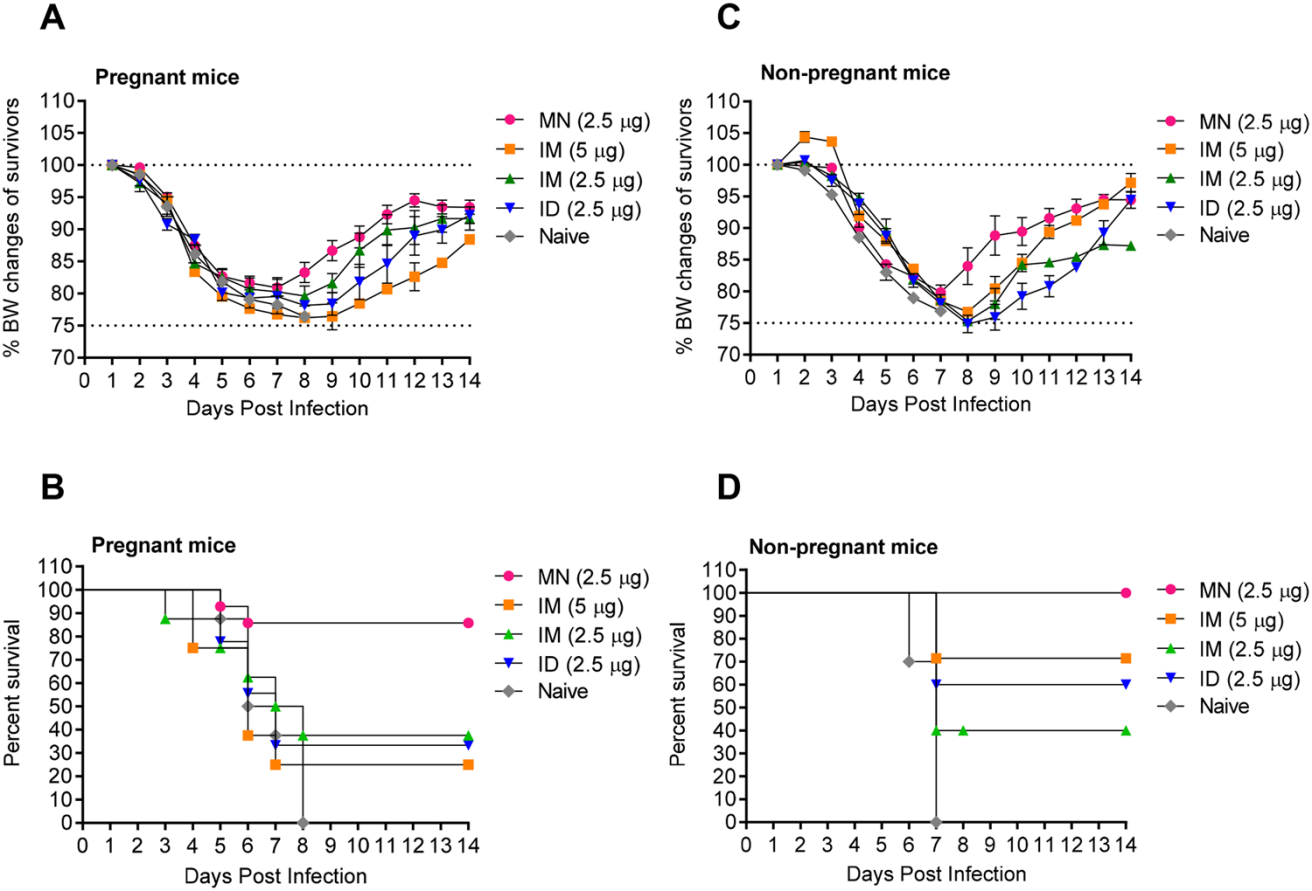
Protective immunity after lethal challenge with homologous virus of mice immunized during pregnancy. Immunized groups were challenged with mouse adapted A/Brisbane/59/07 (H1N1) virus 4 weeks after immunization. (**A** and **C**) Body weight changes and (**B** and **D**) survival rates after challenge with 5xLD_50_ of virus were monitored for 14 days. Values are expressed as mean +/− SEM. (n = 5–14). Groups and animal numbers are as described in Fig. 2; *p < 0.05; **p < 0.01.

HAI and neutralizing antibodies are frequently considered as the most reliable immune correlates in predicting protection against influenza infection. Overall, we observed that the production of influenza-specific HAI and NT titers was dose-dependent in systemic immunization of non-pregnant mice, as seen in the IM vaccinated high (5 μg) and low dose (2.5 μg) groups. The state of pregnancy compromised the production of antibodies by all vaccination route tested; nevertheless, MN immunization with low vaccine dose elicited 4-fold (p < 0.001) and 8-fold (p < 0.0001) higher HAI titers (Fig. 2D) and 3-fold (p < 0.05) and 30-fold (p < 0.0001) higher NT titers than IM immunization with a higher vaccine dose in pregnant and non-pregnant mice respectively, demonstrating significant dose sparing (Fig. 2E) (Supplementary Table 1).

### Skin immunization with MNs conferred complete protection against lethal homologous seasonal influenza virus challenge in both pregnant and non-pregnant mice

Thirty days after immunization, which corresponded to about 21 days after delivery, the mothers were challenged with 5xLD_50_ of homologous influenza virus. All infected animals displayed signs of disease including ruffled hair, hunched posture, and body weight loss; however, pregnant and non-pregnant mice immunized with MNs lost significantly less body weight and demonstrated earlier signs of recovery compared to the groups immunized through traditional routes (Fig. 3A and C). Superior protection of the MN group was also reflected in survival rates. MN-immunized pregnant mice demonstrated 93% survival, significantly higher than the other vaccinated pregnant groups. Mice immunized IM with 5 or 2.5 µg HA of vaccine or ID with 2.5 µg HA showed 25% (p = 0.0015), 20% (p = 0.0015) and 40% (0.0007) survival respectively (Fig. 3B). Interestingly, survival was not dose-dependent in pregnant animals as observed in the non-pregnant cohort. MN vaccination of non-pregnant mice conferred 100% protection whereas IM vaccination with double the vaccine dose (5 µg HA) conferred reduced survival (86%). IM and ID immunized groups that received 2.5 µg HA exhibited a 43% survival rate (Fig. 3D). We observed a significant correlation between HAI titers and protection in the pregnant cohort (R^2^ = 0.972, p = 0.04), whereas this was not the case in the non-pregnant cohort. The lack of a correlation in the non-pregnant population is indicative of involvement of humoral and cellular immunity in survival. In contrast, pregnant mice rely mainly on humoral responses for protection against a lethal challenge dose of influenza virus.

### Offspring born to MN-influenza vaccinated mothers during pregnancy showed superior passive immunity to pups born to IM or ID mice immunized pregnant mice

**Figure 4:**
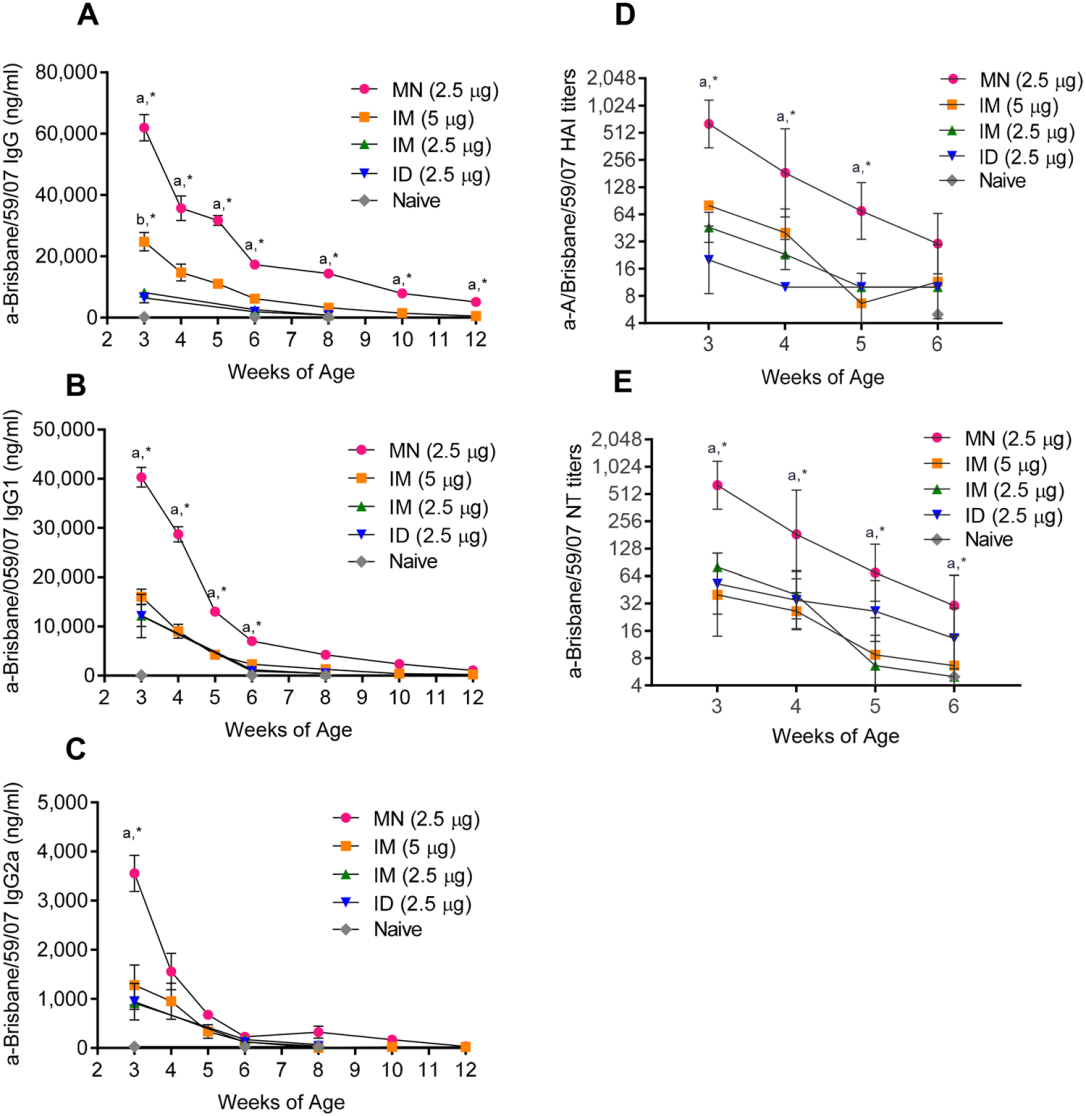
Humoral immune responses in pups born to mothers immunized during pregnancy. Anti-influenza binding antibodies were determined by ELISA in sera collected from pups at weeks 3 to 12 after birth. (**A**) IgG, (**B**) IgG1 and (**C**) IgG2a antibody titers [MN (2.5 ug), n = 10; IM (5 µg), n = 10; IM (2.5 µg), n = 5; ID (2.5 µg), n = 5; Naïve, n = 5]. Values are expressed as mean ±SEM. (**D**) Hemagglutination inhibition (HAI) and (**E**) neutralizing antibody (NT) titers in sera collected 3, 4, 5, and 6 weeks after birth (n = 5 per group). HAI and NT values are expressed as geometric mean with ±95% confidence interval. ^a^[MN *vs* IM (5 μg), IM (2.5 μg), ID (2.5 μg)]. ^b^[IM(5 μg) *vs* IM (2.5 μg), ID (2.5 μg)]. *p < 0.05. Statistical significance was determined using the Bonferroni-Dunn method, with alpha = 0.05.

According to CDC recommendations infants younger than 6 months of age should not receive the influenza vaccine. However, immunized mothers can transfer antibodies to their offspring during gestation through the placenta or post-birth through breast milk^39, 40^. Influenza-specific antibody levels in mouse pups were correlated with the humoral responses elicited in the mothers. At the end of the weaning period (week 3), the influenza-specific IgG, IG1, and IgG2A antibody titers were at least 2.5 times higher (p < 0.05) in pups born to MN-vaccinated mice compared to mice born to mothers vaccinated with either the same vaccine dose IM or ID or two-fold higher dose administered IM (Fig. 4A,B and C) (Supplementary Table 2). In all groups, influenza-specific antibody titers decreased over time, but the MN offspring had the longest lasting passive immunity. From weeks 4 through 12, IgG and IgG1 antibody titers in the MN offspring ranged from 2 to 10-fold higher than pups born to mothers with the higher IM dose (Fig. 4A and B)

The HAI and neutralizing antibody levels were at least 8-fold higher (p < 0.001) in pups from MN-immunized mothers for the first 3 weeks after birth compared to the remaining groups (Fig. 4D and E) (Supplementary Table 2). Six week old offspring from MN vaccinated mothers had HAI and NT titers close to 40 suggestive of protective immunity whereas mice born to mothers immunized IM or ID with a low or high vaccine dose had titers around 10. Interestingly, we noted a dose-dependent response in the production of HAI antibodies in the IM vaccinated mothers’ offspring until week 4 (p = 0.015) (Fig. 4D).

### Microneedle immunization of pregnant mice resulted in improved protection of their offspring compared to IM or ID vaccination

**Figure 5:**
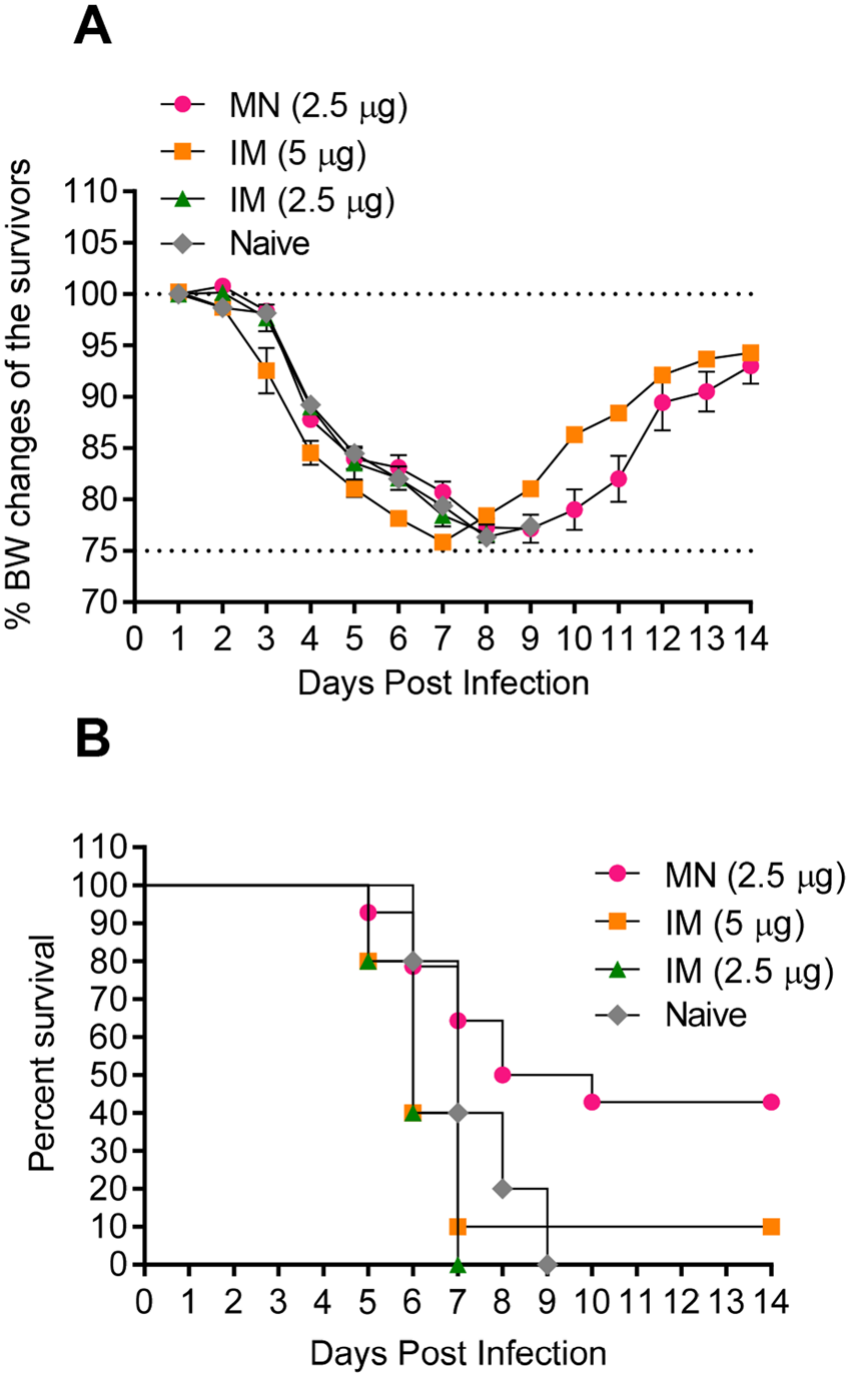
Protective immunity after challenge with A/Brisbane/59/07 virus of offspring born to mothers immunized during pregnancy. Offspring were challenged with 3xLD_50_ of mouse adapted A/Brisbane/59/07 virus 6 weeks after birth. (**A**) Body weight changes were monitored for 14 days (**B**) Survival rates. Values are expressed as mean +/− SEM. Groups and numbers are as described in Fig. 4.

The protective efficacy of maternal anti-influenza IgG antibodies transferred to fetuses via the placenta or to the newborns via maternal milk was tested in 6 week-old pups following challenge with 5xLD_50_ mouse adapted homologous influenza virus (Fig. 5A,B). Although the percent changes of BW over the initial period of 7–8 days (weight loss) were not as prominent for the various offspring groups as in the adult population, the averages at any given time point show that the MN group benefited most from the vaccination (Fig. 5A). It should be mentioned here that the average percentage changes in body weights are affected by the numbers of surviving mice. The highest survival rates were observed in pups from MN-immunized mothers (50% survival) and correlated well with the levels of neutralizing antibodies (Fig. 5B and D). These rates were significantly higher than those observed in pups of pregnant mice immunized intramuscularly with 5 µg or 2.5 µg of HA, which showed survival rates of 10% (p = 0.03) and 0% (p = 0.05) respectively. There was a strong correlation between hemagglutination inhibition antibody titers (HAI) and percent survival (R^2^ = 0.986, p = 0.01). These data confirm the superiority of MN vaccination, since the passive immunity observed in the offspring of vaccinated mothers was robust and long-lived, with protective antibodies persisting at least up to 6 weeks after birth.

## Discussion

During pregnancy, the immune system adapts to tolerate a genetically different fetus as a foreign entity. Dramatic alterations in pro- and anti-inflammatory cytokine profiles and changes in Th1 and Th2 responses exert a suppressive effect on a mother’s immune system pre-disposing her to increased susceptibility to infection and endangering the outcome of pregnancy^41^. Vaccination of pregnant women has a “two-in-one” benefit of protecting both the mother and infant. Mothers can transfer protective antibodies through placenta or through breast milk to the offspring, providing protection in infants until they are old enough to be immunized^39, 40^. Several reports showed that influenza vaccination of pregnant mothers is associated with improved pregnancy outcomes^11, 42–44^ and is up to 63% effective in preventing laboratory confirmed influenza infection in infants^45^.

In this study we investigated the potential of influenza skin immunization with MNs as a platform to improve host immune responses in pregnancy that could in turn effectively protect newborns by passive transfer through the placenta or breast-feeding milk. We used the BALB/c mouse model that shares the same hemochorial placentation as humans, and although the gestation period in BALB/c mice is short (approximately 20–21 days) we were able to time their pregnancies and immunize at mid-gestation simulating human immunizations, as the second or last trimester is the preferred time for pregnant women to receive influenza vaccine. We did not observe any adverse effects of MN vaccination in pregnant mice. No physical marks were left on the skin at the site of immunization, no behavioral changes were noted, the body weight increase was constant, and premature deliveries were not observed suggesting that the MN application did not affect the pregnant females or their fetuses.

We first compared the magnitude of humoral immune responses by assessing the levels of functional antibody titers, including HAI and neutralizing antibody titers, which are considered correlates of protection, in the sera from MN, IM and ID vaccinated pregnant and non-pregnant mice. Although pregnant vaccinated mice had significantly lower humoral immune responses than their non-pregnant counterparts, we found that delivery of influenza vaccine with MNs could confer superior protective immune responses compared to IM or ID immunization in both cohorts. In addition to the induction of robust immune responses, significant dose sparing was observed when using the MN method of vaccine delivery. Adult non-pregnant and pregnant mice that received a single dose of 2.5 μg HA A/Brisbane/59/07 H1N1 subunit vaccine delivered with MN produced higher anti-influenza antibody titers than mice having received a 2-fold higher dose of the vaccine via the IM route.

Complete protection against lethal infection (5xLD_50_) from homologous mouse adapted virus was observed in pregnant and non-pregnant animals vaccinated with MN, whereas animals vaccinated via IM or ID administration with 2.5 or 5 μg HA of A/Brisbane/59/07 during gestation were not protected and non-pregnant IM or ID vaccinated animals were partially protected. These findings are similar to those observed in our previous study of tetanus toxoid vaccination using MNs in pregnant mice^35^.

Previous studies showed that pups born to mice that were vaccinated IP or IM during pregnancy were protected from lethal challenge with influenza virus^19, 20^. In addition, pups born to non-immunized mice but cross-fostered by immunized mothers were also protected, suggesting that protective immunity can be transferred through breast milk^19, 20^. In the present study, we demonstrated that pups born to mothers immunized using MNs encapsulating subunit influenza vaccine had higher levels of specific anti-influenza antibodies in sera than mice that were born to mothers immunized with a two-fold higher dose of the same vaccine via the IM route, The superiority of the MN route was more prominent when compared to the ID or IM vaccination route of mothers that received the same vaccine dose. The highest antibody titers were observed in 3 week-old pups that were still housed with the mothers; a gradual decrease was observed with time following separation from mothers. These results are consistent with reports showing that protective immunity decreases with time after separation from mothers^19, 20^. Pups born to MN immunized mothers maintained significantly higher antibody titers at any tested time and, for up to nine weeks after weaning than pups born to intramuscularly immunized mice. Higher survival rates after lethal challenge with homologous virus three weeks after weaning were observed in pups born to MN immunized mice.

These data demonstrate the benefit of developing a simple-to-administer vaccination method that overcomes the lack of adequate health care in developing countries while achieving protective immunity superior or at least equivalent to conventional immunization. Considering the advantages of the MN technology (thermostability, self-administration, safety, no biohazardous sharps, dose sparing, and robust induction of immune responses at lower doses of vaccine), we believe that a MN vaccination strategy is an attractive option for vaccination of pregnant women, which can aid in achieving the third United Nations Sustainable Development Goal which aims to reduce mortality of children younger than 5 and to reduce maternal mortality^46^.

## Materials and Methods

### Cells and virus stocks

Madin-Darby canine kidney (MDCK) cells (CCL 34, ATCC, Manassas, VA) were cultured in Dulbecco’s Modified Eagle’s Medium (Mediatech, Herndon, VA) containing 10% fetal bovine serum (Hyclone, Thermo Scientific, Rockford, IL). Influenza virus stocks (A/Brisbane/59/07 (H1N1)) were propagated in MDCK cells. Mouse-adapted virus was serially passaged in BALB/c mouse lungs, and viral stock titers were determined by plaque assay^47^. The LD_50_ was determined using the Reed-Munch formula^48^. The hemagglutination activity was determined using turkey red blood cells (LAMPIRE, Pipersville, PA)^49^.

### MNs containing influenza vaccine

MNs were manufactured in a two-step micro-molding process described previously^35^ and stored with a desiccant in individual sealed pouches until use. The bending and brittleness of the microneedles was visually evaluated under the microscope before and after insertion of MN into pig skin explants (not shown). The antigen dose was adjusted to 2.5 μg per patch and mounted onto a 1 cm^2^ paper disk for application.

### Delivery efficiency of MNs

To determine antigen delivery efficiency, MNs were prepared according to the protocol described by Vassilieva^50^. Freshly prepared MNs encapsulating A/Brisbane/59/2007 monovalent vaccine were cut in halves. One half was manually applied to mouse skin, firmly held in place for 1 minute and left on skin for an additional 9 minutes while the other, unused half was kept as a control. The hemagglutinin (HA) content was measured in both used and unused halves with SRID assay.

### Single Radial Immunodiffusion (SRID) assay

Antigen content in each MN was quantified in triplicate via SRID as described previously^50^.

### Animals

Eight-week-old male and female BALB/c mice were purchased from Harlan Laboratories (Dublin, VA). All mice were bred and housed in the Emory University Whitehead Animal Facility. Immunizations were performed in a Biosafety Level 1 facility, and influenza challenge studies were conducted in a Biosafety Level 2 facility at Emory University (Atlanta, GA). All animal breeding, vaccination, and infection experiments were performed in accordance with relevant guidelines and regulations and approved by the Institutional Animal Care and Use Committee (IACUC) at Emory University.

### Breeding protocol

Breeding cages were set up with three females in proestrus or estrus and one male for 3 days^51, 52^. The timing of pregnancies was determined by the presence of copulation plugs and body weight increases in female mice after mating^35^.

### Immunization and sample collection

**Figure 6:**
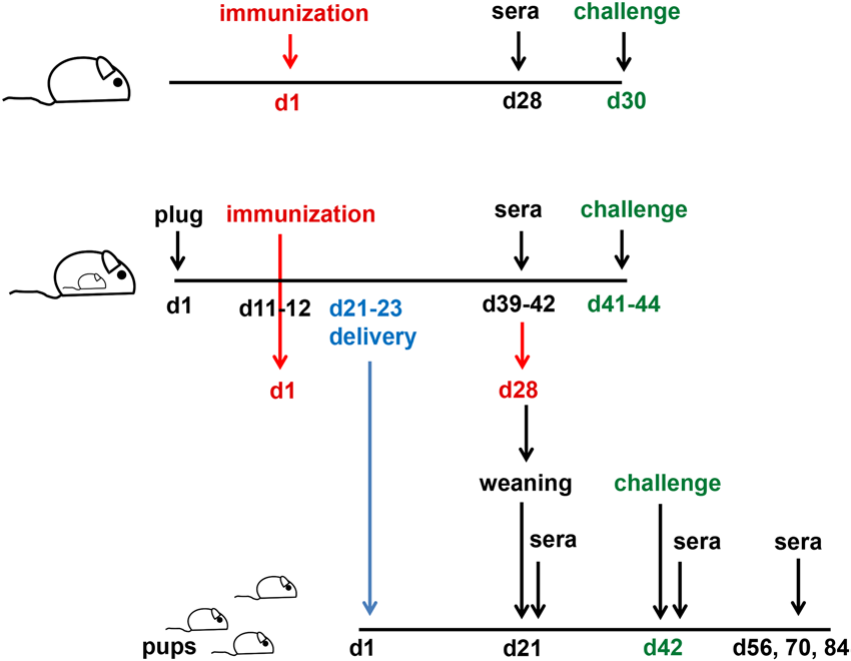
Schematic presentation of animal vaccination and challenge. Pregnant BALB/c mice were immunized at mid gestation with A/Brisbane/59/07 H1N1 vaccine via the intramuscular (IM), intradermal (IM), or cutaneous route using fish gelatin microneedles encapsulating the vaccine (MN). Non-pregnant female mice were also immunized with the same vaccine via the same routes. On day 28 after immunization mice were bled and then challenged with homologous virus. The pups were weaned three weeks after birth and bled on weeks 3, 4, 5, 6, 8, 10, and 12. A group of 6 week old pups was challenged with A/Brisbane/59/07 H1N1 virus 6 weeks after birth.

Female pregnant and non-pregnant mice were immunized cutaneously with MN, intramuscularly (IM) or intradermally (ID) at mid-gestation (days 11–13) as outlined in Fig. 6. For MN immunization, mouse skin was exposed by trimming hair and 1 minute application of depilatory cream (Nair, Church and Dwight, Ewing, NJ) on the caudal area on the dorsal side. MNs were manually inserted with constant pressure into the skin for 1 minute and left in place for 10 minutes to allow microneedle dissolution and delivery of 2.5 µg HA antigen. For IM immunizations, 5 or 2.5 µg HA antigen was diluted in PBS and injected into the biceps femoris in the hind leg of the mice. Blood was collected submandibularly at 4 weeks post-immunization and serum was stored at −20 °C until analysis. The pups were weaned 3 weeks after birth (day 21) and bled at weeks 3, 4, 5, 6, 8, 10 and 12 after birth (Fig. 6).

### Humoral immune responses

Influenza-specific antibody serum concentrations were quantified via ELISA in Nunc 96-well Maxisorb plates (Rochester, NY) coated with 100 ng total protein of seasonal A/Br/59/07 vaccine per well^53^. Hemagglutination inhibition **(**HAI) titers were determined using turkey red blood cells (LAMPIRE, Pipersville, PA) WHO protocol^49, 54^. Sera from vaccinated mice was heat inactivated and neutralizing potency was determined in a microneutralization assay using 100 TCID_50_/well of H1N1 A/Brisbane/59/07 virus^17^.

### Challenge studies of adult and young mice

Adult mice and their offspring were lightly anesthetized under 2 ml isoflurane in a 500-ml enclosed beaker and infected intranasally with 30 μl 5xLD_50_ of mouse adapted A/Brisbane/59/07 virus at 6 weeks after birth. The LD_50_ was calculated separately for adult and young mice. The animals were monitored for 14 days for body weight changes, fever, hunched posture, and mortality. Weight loss exceeding 25% of the starting body weight was used as the experimental end point, at which mice were euthanized according to relevant IACUC guidelines and regulations.

### Statistics

Statistical significance between experimental groups was calculated via Mann-Whitney two-tailed unpaired non-parametric *t*-tests with alpha = 0.05. Antibody assays (ELISA, HAI, microneutralization) were duplicated except where noted otherwise. HAI and NT titers were analyzed as log_2_ titers. For survival curves, statistics were calculated using a Log-rank (Mantel-Cox) test. Non-linear regression analyses were performed to determine the IC_50_ (95% confidence interval). A *p* value ≤ 0.05 was considered significant. Pearson correlation coefficients were applied to correlate humoral responses to survival.

### Data Availability

All data generated or analyzed during this study are included in this published article.

## Electronic supplementary material

Supplemental Tables
