# Supplementary material for: Microneedle patch delivery of influenza vaccine during pregnancy enhances maternal immune responses promoting survival and long-lasting passive immunity to offspring
Source: Sci Rep. 2017 Jul 18;7:5705. doi: 10.1038/s41598-017-05940-7 (PMC5515933; doi:10.1038/s41598-017-05940-7)
Supplement: Supplementary file 1 — Supplemental Tables [file 41598_2017_5940_MOESM1_ESM.doc]

**Microneedle patch delivery of influenza vaccine during pregnancy enhances maternal immune responses**

**promoting survival and long-lasting passive immunity to offspring**

E. Stein Esser^1,a^Joanna A. Pulit-Penaloza^1, a^, Haripriya Kalluri^2^, , Devin McAllister^2^ , Elena V. Vassilieva^1^,

Elizabeth Q. Littauer^1^, Nadia Lelutiu^1^, Mark R. Prausnitz^2^, Richard W. Compans^1^, and Ioanna Skountzou^1^,*

^1^Department of Microbiology & Immunology and Emory Vaccine Center, Emory University School of Medicine, Atlanta, GA 30322

^2^School of Chemical and Biomolecular Engineering, Georgia Institute of Technology, Atlanta, GA 30332

*; to whom correspondence should be addressed: [iskount@emory.edu](mailto:iskount@emory.edu)

1518 Clifton Road NE, Claudia Nance Rollins Building, Atlanta, GA 30322

^a^; these authors contributed equally to this work

**Supplementary Tables:** Summary of fold-changes in humoral responses and survival rates elicited in pregnant mice and their offspring as well as their non-pregnant controls induced intramuscularly or cutaneously.

| **Suppl. Table 1. Summary of fold changes and statistical differences in humoral responses in pregnant or non-pregnant mice immunized via intramuscular or transcutaneous routes.** | | | | | | | | | | | | |
| --- | --- | --- | --- | --- | --- | --- | --- | --- | --- | --- | --- | --- |
|  | MN (2.5 µg) / IM (5 µg) | | | | MN (2.5 µg) / IM (2.5 µg) | | | | MN (2.5 µg) / ID (2.5 µg) | | | |
|  | Pregnant | P-value | Non-Pregnant | P-value | Pregnant | P-value | Non-Pregnant | P-value | Pregnant | P-value | Non-Pregnant | P-value |
| IgG | 3.2 | <0.0001 | 7.5 | <0.0001 | 5.1 | <0.0001 | 10.8 | <0.0001 | 4.1 | <0.0001 | 10.7 | <0.0001 |
| IgG1 | 2.8 | <0.0001 | 5.3 | <0.0001 | 7.3 | <0.0001 | 15.7 | <0.0001 | 5.7 | <0.0001 | 13.1 | <0.0001 |
| IgG2a | 4.3 | 0.0074 | 25.5 | <0.0001 | 6.3 | 0.0006 | 10.0 | <0.0001 | 6.9 | 0.0005 | 10.7 | <0.0001 |
| HAI | 4.3 | <0.0001 | 10.2 | <0.0001 | 8.0 | <0.0001 | 11.3 | 0.0009 | 4.9 | <0.0001 | 14.9 | 0.0007 |
| NT | 2.7 | 0.0066 | 21.1 | <0.0001 | 2.2 | 0.0021 | 20.4 | <0.0001 | 2.9 | 0.0113 | 23.4 | <0.0001 |
| *Fold changes were calculated by dividing mean values. P values were calculated using Student's t test. P values ≥ 0.05 were considered not statistically significant (ns).* | | | | | | | | | | | | |

| **Suppl. Table 2. Summary of survival rates and statistical differences in pregnant and non-pregnant mice immunized via intramuscular or transcutaneous routes and challenged with 5xLD50.** | | | | |
| --- | --- | --- | --- | --- |
|  | **Pregnant** | | **Non-Pregnant** | |
|  | Survival (%) | Comparison with MN  (p-value) | Survival (%) | Comparison with MN  (p-value) |
| MN (2.5 µg) | 92.9 | --- | 100 | --- |
| IM (5 µg) | 25 | 0.005 | 71.4 | 0.21 |
| IM (2.5 µg) | 20 | 0.002 | 40 | 0.05 |
| ID (2.5µg) | 42.9 | 0.013 | 40 | 0.05 |
| Naive | 0 | 0.0001 | 0 | 0.0008 |
| *P values were calculated using Mantel-Cox test. P values ≥ 0.05 were considered not statistically significant (ns).* | | | | |

| **Suppl. Table 3. Summary of fold changes and statistical differences in humoral responses in offspring of mice immunized via intramuscular or transcutaneous routes.** | | | | | | | |
| --- | --- | --- | --- | --- | --- | --- | --- |
| Antibodies | Week | MN (2.5 µg) /IM (5 µg) | | MN (2.5 µg) /IM (2.5 µg) | | MN (2.5 µg) /ID (2.5 µg) | |
|  |  | Fold | p value | Fold | p value | Fold | p value |
| IgG | 3 | 2.5 | <0.0001 | 7.6 | <0.0001 | 9.6 | <0.0001 |
|  | 6 | 2.8 | <0.0001 | 6.8 | <0.0001 | 9.0 | <0.0001 |
|  | 8 | 5.9 | <0.0001 | 25.6 | <0.0001 | 24.8 | <0.0001 |
| IgG1 | 3 | 2.5 | <0.0001 | 3.3 | <0.0001 | 3.3 | 0.0014 |
|  | 6 | 3.0 | <0.0001 | 5.9 | <0.0001 | 7.8 | <0.0001 |
|  | 8 | 3.3 | 0.0040 | 10.8 | 0.0007 | 9.9 | 0.0007 |
| IgG2a | 3 | 2.8 | 0.0007 | 3.9 | <0.0001 | 3.8 | 0.0004 |
|  | 6 | 1.8 | ns | 1.3 | ns | 1.9 | ns |
|  | 8 | 469.2 | 0.0266 | 4.6 | 0.0680 | 7.8 | 0.0462 |
| HAI | 3 | 8.0 | 0.0007 | 13.9 | <0.0001 | 32.0 | <0.0001 |
|  | 6 | 2.6 | ns | 3.0 | 0.0161 | 3.0 | 0.0161 |
| NT | 3 | 16.0 | 0.0006 | 8.0 | 0.0007 | 12.1 | <0.0001 |
|  | 6 | 4.6 | 0.0046 | 2.6 | ns | 0.9 | ns |
| *Fold changes were calculated by dividing mean values. P values were calculated using Student's t test. P values ≥ 0.05 were considered not statistically significant (ns).* | | | | | | | |

| **Suppl. Table 4. Summary of survival rates and statistical differences in offspring of mice immunized via intramuscular or transcutaneous routes challenged with 3xLD50.** | | |
| --- | --- | --- |
|  | Survival (%) | Comparison with MN  (p-value) |
| MN (2.5 µg) | 42.9 | --- |
| IM (5 µg) | 10 | 0.020 |
| IM (2.5 µg) | 0 | 0.019 |
| Naive | 0 | 0.035 |
| *P values were calculated using Mantel-Cox test. P values ≥ 0.05 were considered not statistically significant (ns).* | | |
